# Auxin and ABA join forces through bZIP23 to promote rice seed vigor

**DOI:** 10.1093/plcell/koag240

**Published:** 2026-08-06

**Authors:** Jiajun Wang, Wenjing Liu

**Affiliations:** Assistant Features Editor, the Plant Cell, American Society of Plant Biologists; School of Life Sciences, Xiamen Key Laboratory of Plant Genetics, Xiamen University, Xiamen 361102, China; School of Life Sciences, Xiamen Key Laboratory of Plant Genetics, Xiamen University, Xiamen 361102, China

Seed vigor is an important agronomic trait, typically referring to the ability of seeds to germinate (germination vigor) and to maintain this vigor during storage, which is of great significance for seed quality and germplasm conservation. High-vigor seeds improve planting efficiency by ensuring rapid and uniform seedling establishment, thereby reducing seed input requirements and minimizing the need for reseeding and thinning. Seed vigor generally declines during storage, and the rate of deterioration is influenced not only by the physiological status of the seed itself but also by environmental factors, particularly storage temperature and seed moisture content (Zhao et al. 2021). Although both abscisic acid (ABA) and auxin signaling have been implicated in seed vigor regulation, the molecular mechanisms by which these 2 pathways interact remain poorly understood. In new work, **Xiao-Hui Feng and colleagues** (Feng et al. 2026) revealed that auxin and ABA pathways converge on bZIP23 to promote rice seed vigor.

*bZIP23* encodes a member of the basic leucine zipper (bZIP) transcription factor family and plays a central role in ABA signaling. Previous studies have shown that bZIP23 interacts with and is phosphorylated by Osmotic Stress/ABA-Activated Protein Kinase 2 (SAPK2), a homolog of SnRK2 protein kinases. Phosphorylation of bZIP23 enhances its transcriptional activity, allowing it to modulate the expression of genes involved in ABA signaling and biosynthesis (Zong et al. 2016). Consistent with its role in ABA signaling, *bzip23* mutants are less sensitive to ABA during seed germination, whereas *bZIP23*-overexpressing plants show increased sensitivity, exhibiting greater ABA-mediated inhibition of seedling growth (Xiang et al. 2008).

In addition, *bZIP23* is induced by ABA treatment and accelerated seed aging and promotes seed vigor by activating the peroxiredoxin gene *PER1A* (Wang et al. 2022). Notably, several ABA signaling genes, including *SAPK1*, are also upregulated during accelerated seed aging, prompting Feng et al. to investigate the role of SAPK1 in seed vigor regulation. First, the authors found that during accelerated seed aging, *SAPK1* transcript levels were upregulated in the high-vigor cultivar Kasalath but remained unchanged in the low-vigor Jigeng88. CRISPR-generated *sapk1* mutants exhibited significantly reduced germination and seedling emergence rates following accelerated seed aging, whereas seed-specific overexpression of *SAPK1* substantially improved seed performance after aging treatment.

Following accelerated aging, *sapk1* mutant seeds accumulated higher levels of hydrogen peroxide in the endosperm and exhibited reduced cellular viability compared with the control. In contrast, seed-specific overexpression of *SAPK1* produced the opposite phenotype, suggesting that SAPK1 promotes seed vigor by alleviating oxidative stress. Given the critical role of the bZIP23–PER1A detoxification module in maintaining seed viability, the authors next examined whether SAPK1 regulates bZIP23. They found that SAPK1 physically interacts with and phosphorylates bZIP23 both in vitro and in vivo. Mass spectrometry analysis identified 4 SAPK1-dependent phosphorylation sites on bZIP23. Simultaneous substitution of these sites with alanine markedly reduced bZIP23-mediated activation of *PER1A*, whereas phosphomimetic substitutions significantly enhanced its transactivation activity. Consistent with these molecular findings, phosphomimetic bZIP23 enhanced seed vigor, whereas the phosphorylation-deficient bZIP23-4A variant failed to do so. Genetically, bZIP23 overexpression partially rescued the reduced germination and seedling emergence rates, as well as the elevated hydrogen peroxide accumulation, observed in the *sapk1* mutant following accelerated seed aging.

The *bZIP23* promoter contains 2 auxin response elements (AuxREs), prompting the authors to investigate whether auxin response factors regulate *bZIP23* expression. They found that both *OsARF12* and *bZIP23* were induced during accelerated seed aging and that the induction of *bZIP23* depended on OsARF12. Moreover, OsARF12 directly bound to the *bZIP23* promoter and activated its transcription. Phenotypic analyses demonstrated that OsARF12 positively regulates seed vigor during accelerated aging. Genetic evidence further placed bZIP23 downstream of OsARF12, as overexpression of *bZIP23* partially rescued the seed vigor defects of *osarf12* mutants.

Interestingly, the entire canonical auxin signaling pathway, consisting of OsTIR1, OsIAA10, and OsARF12, is required for seed vigor regulation. Genetic analyses showed that OsTIR1 promotes seed vigor during accelerated aging, whereas OsIAA10 acts downstream of OsTIR1 to repress seed vigor. OsARF12 functions downstream of OsIAA10 and positively regulates seed vigor.

Together, the authors demonstrated that the canonical OsTIR1–OsIAA10–OsARF12 auxin signaling pathway promotes seed vigor during accelerated aging by inducing *bZIP23* expression while SAPK1, a core protein kinase in the ABA signaling pathway, enhances seed vigor by phosphorylating bZIP23 and increasing its transactivation of *PER1A* (see Fig. 1). Acting as a central integration node for auxin and ABA signaling, bZIP23 serves as a molecular bridge that coordinates hormonal crosstalk to maintain seed vigor during aging. These findings provide a mechanistic framework for understanding how multiple hormonal pathways converge to regulate seed longevity and vigor.

**Figure 1:**
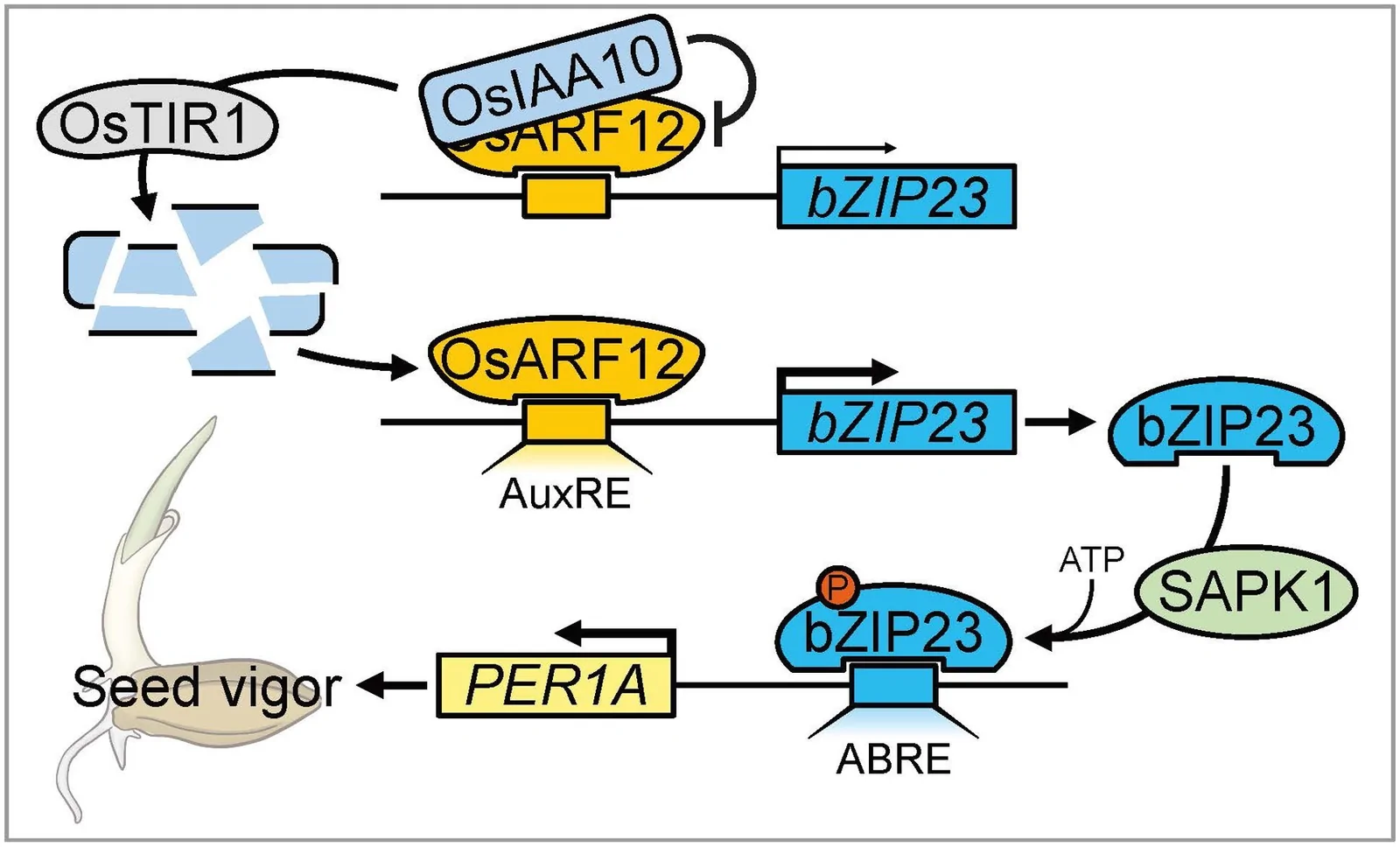
A model illustrating the convergence of auxin and ABA signaling on bZIP23 to regulate seed vigor. Auxin promotes *bZIP23* transcription via the canonical OsTIR1–OsIAA10–OsARF12 signaling pathway, whereas SAPK1 phosphorylates bZIP23 to enhance its transcriptional activation of *PER1A*, thereby positively regulating seed vigor. Reprinted from Feng et al. (2026), Figure 8h.

## Recent related articles in *The Plant Cell*

- Zhang et al. (2025) reveal that natural variation in the *ZmPIMT1* promoter enhances seed aging tolerance by increasing *ZmPIMT1* expression, thereby promoting the repair of damaged PABP2 proteins and maintaining seed vigor in maize.
- Qiu et al. (2025) showed that the auxin signaling component OsIAA7 restricts rice grain enlargement by promoting OsGSK2-mediated phosphorylation and degradation of OsBZR1, thereby linking auxin and brassinosteroid signaling in the regulation of grain size. In an accompanying *In Brief*, Wang (2025) highlighted this work as an example of how auxin signaling coordinates crosstalk with other phytohormone pathways to regulate important agronomic traits.
- Tang et al. (2025) demonstrate that the evening complex proteins ELF3, ELF4, and LUX antagonize ABI3- and ABI5-mediated ABA signaling, thereby linking circadian clock cues to the temporal regulation of seed germination under stress conditions.
